# Microstructural Abnormalities Were Found in Brain Gray Matter from Patients with Chronic Myofascial Pain

**DOI:** 10.3389/fnana.2016.00122

**Published:** 2016-12-20

**Authors:** Peng Xie, Bangyong Qin, Ganjun Song, Yi Zhang, Song Cao, Jin Yu, Jianjiang Wu, Jiang Wang, Tijiang Zhang, Xiaoming Zhang, Tian Yu, Hong Zheng

**Affiliations:** ^1^Department of Anesthesiology, The First Affiliated Hospital of Xinjiang Medical UniversityUrumqi, China; ^2^Department of Anesthesiology, Zunyi Medical UniversityZunyi, China; ^3^Department of Radiology, Zunyi Medical UniversityZunyi, China; ^4^Guizhou Key Laboratory of Anesthesia and Organ Protection, Zunyi Medical UniversityZunyi, China; ^5^Department of Anatomy and Cell Biology, University of Kansas Medical Center, Kansas CityKS, USA

**Keywords:** myofascial trigger points, chronic pain, gray matter, microstructural abnormalities, diffusion kurtosis imaging

## Abstract

Myofascial pain, presented as myofascial trigger points (MTrPs)-related pain, is a common, chronic disease involving skeletal muscle, but its underlying mechanisms have been poorly understood. Previous studies have revealed that chronic pain can induce microstructural abnormalities in the cerebral gray matter. However, it remains unclear whether the brain gray matters of patients with chronic MTrPs-related pain undergo alteration. In this study, we employed the Diffusion Kurtosis Imaging (DKI) technique, which is particularly sensitive to brain microstructural perturbation, to monitor the MTrPs-related microstructural alterations in brain gray matter of patients with chronic pain. Our results revealed that, in comparison with the healthy controls, patients with chronic myofascial pain exhibited microstructural abnormalities in the cerebral gray matter and these lesions were mainly distributed in the limbic system and the brain areas involved in the pain matrix. In addition, we showed that microstructural abnormalities in the right anterior cingulate cortex (ACC) and medial prefrontal cortex (mPFC) had a significant negative correlation with the course of disease and pain intensity. The results of this study demonstrated for the first time that there are microstructural abnormalities in the brain gray matter of patients with MTrPs-related chronic pain. Our findings may provide new insights into the future development of appropriate therapeutic strategies to this disease.

## Background

Myofascial pain syndrome is a common type of chronic pain involving the skeletal muscles and is characterized by the presence of myofascial trigger points (MTrPs). MTrPs are clinically defined as highly localized and cord-shaped sensitive areas in the skeletal muscle that have tension and can be palpated. Stretching, pressing, or contraction of the skeletal muscles may trigger characteristic pain, which is often accompanied by referred pain. MTrPs can be classified into active MTrPs and latent MTrPs. Active MTrPs induce spontaneous pain or motion-induced reactive pain, while latent MTrPs induce pain or discomfort only when pressed (Simons, 2008; Xie et al., 2015). As an illness, MTrPs-related myofascial pain has a prevalence of 21–30% in the general population (Borg-Stein and Iaccarino, 2014). In the United States, over 44 million individuals have developed this disease and collectively require 47 billion dollars in medical expenditures every year (Wheeler, 2004).

Currently, clinical treatment for patients with myofascial pain is usually performed at the site of the MTrPs. However, pain often relapses after treatment targeting MTrPs, and it is difficult to treat completely (Graboski et al., 2005; Cummings and Baldry, 2007; Park et al., 2012). Substantial effort has been dedicated to understanding the pathophysiology of MTrPs, and different theories have been proposed, including the integrated hypothesis (Vázquez-Delgado et al., 2009; Bron and Dommertholt, 2012), the central sensitization theory (Mense, 1994; Xu et al., 2010), and the myoelectrical theory (Ge et al., 2006; Huang et al., 2013). However, whether MTrPs are a peripheral or central phenomenon remains controversial (Fernández-de-las-Peñas and Dommerholt, 2014). Current etiological studies on MTrPs almost exclusively focus on the peripheral pathogenesis route; thus, the brain microstructural alterations in patients with MTrPs-related chronic pain remain unclear.

Previous studies have indicated that chronic pain can induce volume changes in the brain (May, 2008; Kregel et al., 2015; de Kruijf et al., 2016). The brain gray matter is closely associated with the body’s pain perception (Cauda et al., 2014; Emerson et al., 2014). A large body of evidence indicated that the gray matter might exhibit abnormalities under chronic pain status, such as trigeminal neuralgia (Obermann et al., 2013; Li et al., 2016), chronic low back pain (Seminowicz et al., 2011; Fritz et al., 2016), fibromyalgia (Hsu et al., 2009; Robinson et al., 2011), phantom limb pain (Preissler et al., 2013), cyclic menstrual pain (Tu et al., 2010), complex regional pain syndrome (Geha et al., 2008), and migraine (Magon et al., 2015). In addition, the density and volume changes of gray matter in patients with chronic pain are also related to clinical traits (Apkarian et al., 2004; Kuchinad et al., 2007). Thus far, the only study that provided data in this regard revealed that the gray matter abnormalities appeared in patients with temporomandibular disorders combined with masticatory muscle myofascial pain (Younger et al., 2010). It remains unclear whether the gray matter abnormalities are caused by masticatory muscle myofascial pain, by temporomandibular disorders, or by both. More evidence is needed to clarify whether gray matter microstructures undergo alterations in patients with MTrPs-related chronic pain.

Magnetic resonance diffusion imaging technologies, such as Diffusion Tensor Imaging (DTI) and Diffusion Kurtosis Imaging (DKI), are developed recently and are capable of performing non-invasive detection of brain microstructures *in vivo* (Cheung et al., 2012; Egger et al., 2016). DKI is a technological extension of DTI that depicts the non-Gaussian diffusion of water molecules in tissues (Jensen and Helpern, 2010) and features a relatively high accuracy to display microstructures comparing to that of DTI. Therefore, DKI is a well-suited technique to capture changes within complex biological tissues and is particularly adopted to reveal the microstructural changes of brain gray matter (Jensen et al., 2005; Gong et al., 2013, 2014).

In this study, the following hypotheses were tested: (1) the gray matter of patients with MTrPs-related chronic pain may undergo microstructural changes; and (2) such microstructural changes may be associated with the course of the disease and pain intensity. DKI was employed to monitor the differences of gray matter microstructures between healthy volunteers and the patients with MTrPs-related chronic pain. The alterations in the gray matter were then analyzed in relation to pain intensity, the course of disease and age.

## Materials and Methods

This study was approved by Zunyi Medical University and Xinjiang Medical University, Human Ethics Committees. All experimental procedures were conducted in accordance with the Declaration of Helsinki. Prior to the studies, all patients and healthy controls provided signed, informed consent.

### Participants

Thirty seven patients who were treated in the Department of Pain in our hospital for MTrPs-related chronic neck pain from January 2015 to December 2015 were recruited for this study. All subjects were right-handed and aged between 25 and 45 years old. The inclusion criteria were as follows: (1) the presence of at least one active MTrPs in the left upper trapezius (Xie et al., 2015; Barbero et al., 2016); (2) a pain duration longer than 3 months in the middle and upper trapezius and in the surrounding areas according to the subject’s self-description; and (3) a score ≥ 5 on a visual analogue scale (VAS) (0, no pain; 10, the most intense pain). The diagnosis of MTrPs was conducted by a physician in the Department of Pain who had over 10 years of experience in treating myofascial pain syndrome, which was based on the following signs (Gerwin et al., 1997; Myburgh et al., 2011): (1) an apparent taut band with easily exited pain spots could be felt by palpation (hand touching); (2) palpation on pain spots led to local pain and distant referred pain; (3) the involved skeletal muscles had a limited range of motion and might display local muscle twitching upon the quick application of pressure; and (4) a mental tension or a lack of sleep aggravated by pain. The exclusion criteria were as follows: radicular pain, cervical spondylosis, history of rheumatism, history of neurological disorders, whiplash, dislocation of the shoulder, surgical history of the trapezius and the cervical spine, temporomandibular joint disorders, and fibromyalgia. In addition, individuals with pregnancy, mental retardation, mental or emotional disorders, had a body mass index greater than or equal to 30, severe systemic disease, and dysmenorrhea were also excluded. All subjects were asked to cease taking acetaminophen phenolic drugs and non-steroidal anti-inflammatory drugs for 3 days prior to the experiments.

Thirty-six right-handed, age- and gender-matched healthy volunteers were enrolled from the neighboring communities. None of the healthy controls had skeletal muscle pain syndrome or signs of pain in the neck, chest, or upper limbs, or had any active or potential MTrPs in the trapezius. The exclusion criteria of the healthy controls were the same as those of the aforementioned MTrPs patients.

### Image Acquisition

The image acquisition was performed using the Signa 3.0T HDXT high-field NMR platform (GE, USA) with the standard 8-channel head coils. Upon scanning, a sponge mat was used to stabilize the head, and two expansion earplugs were used to shield noise. All participants received brain scans to exclude any organic brain diseases. All images were the results of whole-brain sagittal imaging with the following scanning parameters: TR/TE, 7.8/3; field of view (FOV), 240 mm × 240 mm; matrix dimension, 256 × 256; T1, 450 ms; bandwidth, 31.25 Hz; number of slices, 146; slice thickness, 1 mm; inter-slice gap, 0 mm; number of excitations (NEX), 1; and scanning time, 208 s. In addition, all participants also received neck scanning to exclude cervical diseases.

Single-shot echo-planar imaging was adopted for DKI scanning with the following parameters: TR/TE, 10000/99.3 ms; FOV, 240 mm × 240 mm; matrix dimension, 128 × 128; 3 *b*-values, 0, 1000, and 2000 s/mm^2^ (diffusion gradient being applied in 25 different directions); slice thickness, 4 mm; inter-slice gap, 35 mm; number of slices, 35; and scanning time, 530 s. A total of 1820 images were generated for a whole brain.

Except for one subject, who abandoned the scan due to fear and was therefore excluded from the study, all of the participants successfully finished the scanning.

### Post Processing of Images

First, the original DICOM files were converted into files in the NIFTI format. The images were corrected using the Eddy Current Correction tool in FSL (FMRIB Software Library, Oxford, UK), whereby the artifacts resulting from head movement and gradient coil eddies were removed. Diffusional Kurtosis Estimator (DKE) software was then used to evaluate the DKI parameters including mean kurtosis (MK), axial kurtosis (AK), and radial kurtosis (RK) (Tabesh et al., 2011). Afterward, a voxel-based analysis (VBA) was performed using the Statistic Parametric Mapping (SPM8) software in the MATLAB 2012b operating environment^1^ to spatially normalize and smooth the MK, AK, and RK images. The detailed process was described previously by Sun et al. (2014). In brief, each b0 image was subjected to standardization using the standard EPI template. The standardized images were smoothed using the 6 mm, full-width-at-half-maximum (FWHM) Gaussian kernel, whereby the corresponding b0 template was obtained. The b0 template was then employed to standardize all original b0 images; the standardized data were then written into the MK, AK, and RK images, which were again smoothed using the 6 mm, FWHM Gaussian kernel to improve the signal/noise ratio.

To acquire a gray matter mask, the segmentation algorithm was used in SPM8 software used to divide to the T1 weighted image of each subject into gray matter, cerebrospinal fluid, and white matter. Then, the standard Montreal Neurological Institute space was employed to standardize the partitioned gray matter. Lastly, the 6 mm, FWHM Gaussian kernel was employed to perform averaging and smoothing treatments for all gray matter images. The resulting data were converted into binary masks, which were subsequently analyzed.

### Statistical Analyses

The genders, ages, VAS scores, and pain durations of the MTrPs subjects and healthy controls were statistically analyzed using SPSS 17.0 software (SPSS Inc., Chicago, IL, USA). Gender was analyzed using a Chi-square test, whereas the VAS scores, pain durations, and ages were analyzed using two-sample *t*-tests. *p* < 0.05 was considered statistically significant.

A Voxel-Based Analysis was performed on the SPM8 platform to process all standardized and smoothed MK, AK, and RK images. The DKI parameters (MK, AK and RK values) differences in gray matter between the MTrPs subjects and healthy controls were compared using a two-sample *t*-test. For the voxels with a cluster size above 20, the threshold was set to *p* < 0.001 for uncorrected voxels, and the voxel-level significance was corrected for multiple comparisons using a family wise error (FWE) rate with a threshold of *p* < 0.05. Those gray matter areas that exhibited significant differences in the MK, AK, and PK values between the MTrPs subjects and healthy controls were identified as masks of regions of interest (ROI). These masks were then projected onto the standardized and smoothed images of the 36 MTrPs subjects, whereby the MK, AK, and RK values of the ROI were estimated. The correlation between the pain durations, VAS scores and age of the MTrPs subjects and their MK, AK, and RK values were analyzed using Pearson’s linear correlation coefficient. In addition, we also analyzed the correlation between the age and VAS scores, the VAS scores and pain durations. *p* < 0.05 was considered statistically significant.

## Results

### General Information and Clinical Data of the Subjects

**Table 1** summarizes the general information pertaining to the participants. In this study, 36 patients with MTrPs-related chronic pain (14 males, 22 females) and 36 healthy volunteers (16 males, 20 females) were enrolled. There were no significant differences in age and gender between the MTrPs subjects and healthy controls (*p* > 0.05). The MTrPs lesions in the subjects were all located in the upper left trapezius, and these patients’ VAS scores and pain durations were 6.69 ± 0.92 and 10.47 ± 5.83 months respectively.

**Table 1:** Demographic and clinical data of patients with MTrPs subjects and healthy controls.

	Patients (*n* = 36)	HC (*n* = 36)	*P*-values
Sex (M/F)	14/22	16/20	0.67
Age (years)	44.15 ± 4.57	43.96 ± 4.86	0.91
Location of MTrPs	Left upper trapezius muscle	–	N/A
VAS	6.69 ± 0.92	–	N/A
Duration of pain (months)	10.47 ± 5.83	–	N/A


*MTrPs, myofascial trigger points; HC, healthy controls; M/F, male/female, Data are expressed as means ± SD.*

### Comparison of the DKI Parameters between the MTrPs Subjects and Healthy Controls

**FIGURE 1:**
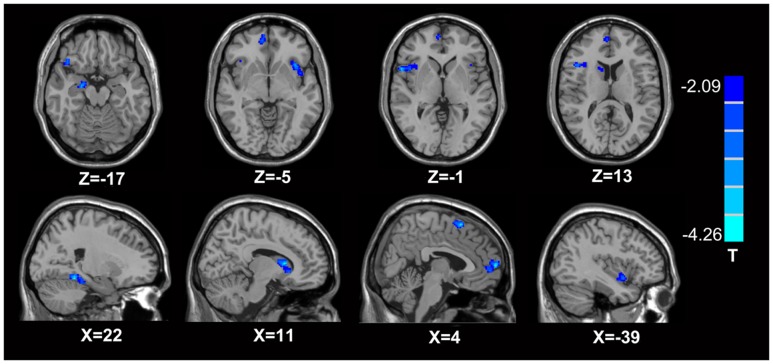
**Brain areas exhibiting significantly lower AK values in MTrPs subjects compared to healthy controls.** The blue zone represents the brain areas with decreased AK values (*p* < 0.05, correction). The colored stripe on the right represents the *T*-value. The left side of the figure represents the anatomical right side, and *vice versa*. MTrPs, myofascial trigger points; AK, axial kurtosis.

**FIGURE 2:**
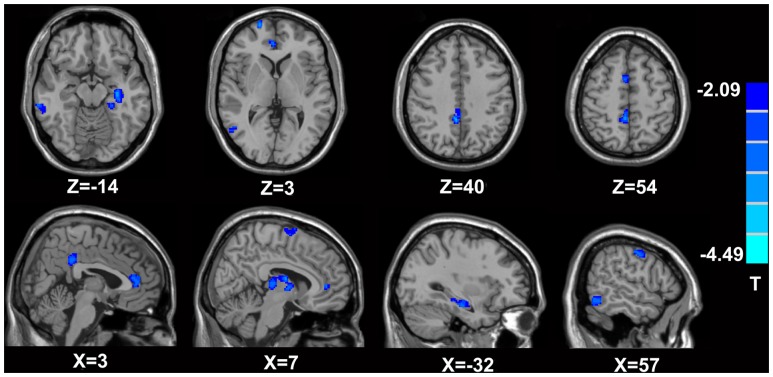
**Brain areas exhibiting significantly lower MK values in MTrPs subjects compared to healthy controls.** The blue zone represents the brain areas with decreased MK values (*p* < 0.05, correction). The colored stripe on the right represents the *T*-value. The left side of the figure represents the anatomical right side, and *vice versa*. MTrPs, myofascial trigger points; MK, mean kurtosis.

**FIGURE 3:**
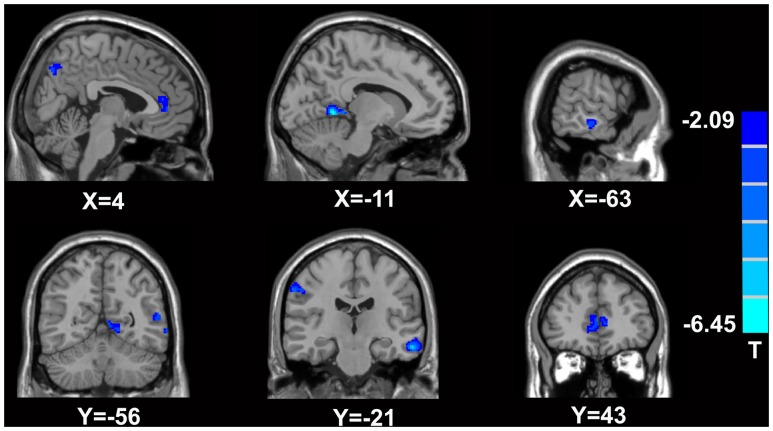
**Brain areas exhibiting significantly lower RK values in MTrPs subjects compared to healthy controls.** The blue zone represents the brain areas with decreased RK values (*p* < 0.05, correction). The colored stripe on the right represents the *T*-value. The left side of the figure represents the anatomical right side, and *vice versa*. MTrPs, myofascial trigger points; RK, radial kurtosis.

In comparison with the healthy controls, the MTrPs subjects displayed significantly lower AK values in the right parahippocampal gyrus (*T*-value: -3.92), right medial prefrontal cortex (mPFC, *T*-value: -4.01), left and right insula (*T*-value: -2.97, -3.04, respectively), right inferior frontal gyrus (*T*-value: -3.56), and right caudate nucleus (*T*-value: -3.22). The degrees of freedom were 70 (**Table 2**; **Figure 1**). Significantly lower MK values were found in the right anterior cingulate cortex (ACC, *T*-value: -3.83), right middle temporal gyrus (*T*-value: -2.36), the left parahippocampal gyrus (*T*-value: -3.50), right superior frontal gyrus (*T*-value: -3.19), right posterior cingulate cortex (PCC, *T*-value: -3.91), and right thalamus (*T*-value: -3.67). The degrees of freedom were 70 (**Table 3**; **Figure 2**). Significantly lower RK values were found in the left lingual gyrus (*T*-value: -6.29), right precentral gyrus (*T*-value: -3.97), left middle temporal gyrus (*T*-value: -5.09), left and right ACC (*T*-value: -2.82, -3.14, respectively), and right precuneus (*T*-value: -2.77). The degrees of freedom were 70 (**Table 4**; **Figure 3**). Nevertheless, no brain area displayed relatively elevated AK, MK or RK values in the MTrPs subjects.

**Table 2:** Brain areas exhibiting significantly lower AK values in MTrPs subjects compared to healthy controls.

Region	Peak MNI coordinates			Number of cluster voxels	Peak *T*-value
			
	*x*	*y*	*z*		
Right parahippocampal gyrus	22	-42	-13	501	-3.92
Right medial prefrontal cortex	4	64	8	923	-4.01
Left insula	-40	11	-5	659	-2.97
Right insula	42	12	1	476	-3.04
Right inferior frontal gyrus	48	16	1	647	-3.56
Right caudate	13	13	13	1021	-3.22


*AK, axial kurtosis; MTrPs, myofascial trigger points; MNI, Montreal Neurological Institute.*

**Table 3:** Brain areas exhibiting significantly lower MK values in MTrPs subjects compared to healthy controls.

Region	Peak MNI coordinates			Number of cluster voxels	Peak *T*-value
			
	*x*	*y*	*z*		
Right anterior cingulate cortex	3	43	3	742	-3.83
Right middle temporal gyrus	57	-56	-14	533	-2.36
Left parahippocampal gyrus	-32	-19	-14	864	-3.50
Right superior frontal gyrus	3	8	54	395	-3.19
Right posterior cingulate cortex	3	-40	36	809	-3.91
Right thalamus	7	-27	6	971	-3.67


*MK, mean kurtosis; MTrPs, myofascial trigger points; MNI: Montreal Neurological Institute.*

**Table 4:** Brain areas exhibiting significantly lower RK values in MTrPs subjects compared to healthy controls.

Region	Peak MNI coordinates			Number of cluster voxels	Peak *T*-value
			
	*x*	*y*	*z*		
Left lingual gyrus	-11	-56	2	1106	-6.29
Right precentral gyrus	58	-21	41	632	-3.97
Left middle temporal gyrus	-63	-21	-17	904	-5.09
Left anterior cingulate cortex	-4	41	1	1002	-2.82
Right anterior cingulate cortex	4	43	5	365	-3.04
Right precuneus	4	-70	42	421	-2.77


*RK, radial kurtosis; MTrPs, myofascial trigger points; MNI: Montreal Neurological Institute.*

### Correlation between Pain Durations, VAS Scores, Age and DKI Parameters in the MTrPs Subjects

**FIGURE 4:**
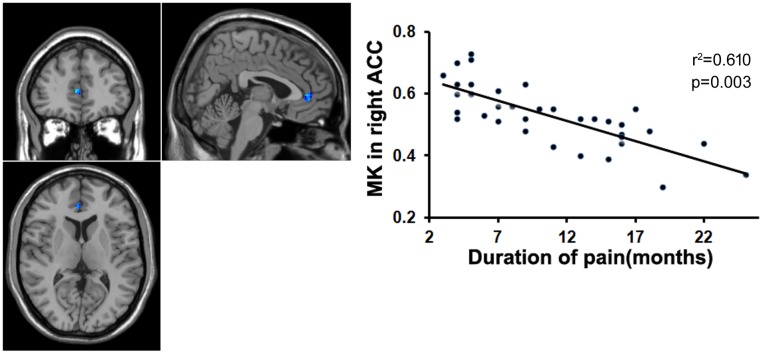
**Correlation analysis between the MK value and the course of disease.** There was significant negative correlation between the MK value in the right ACC and course of disease in patients with chronic myofascial pain.

**FIGURE 5:**
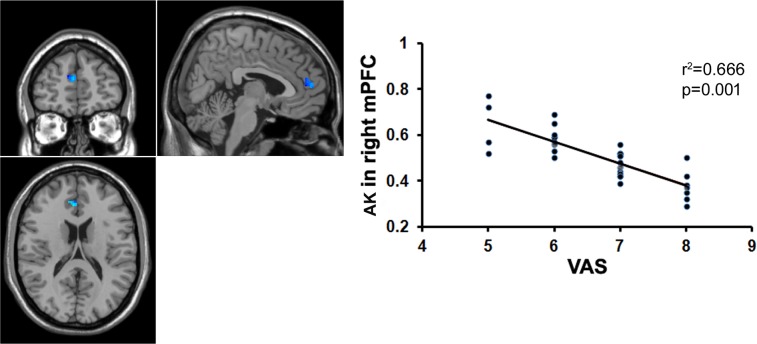
**Correlation analysis between the MK value and the VAS score.** There was significant negative correlation between the MK value in the right ACC and the VAS score in patients with chronic myofascial pain.

**FIGURE 6:**
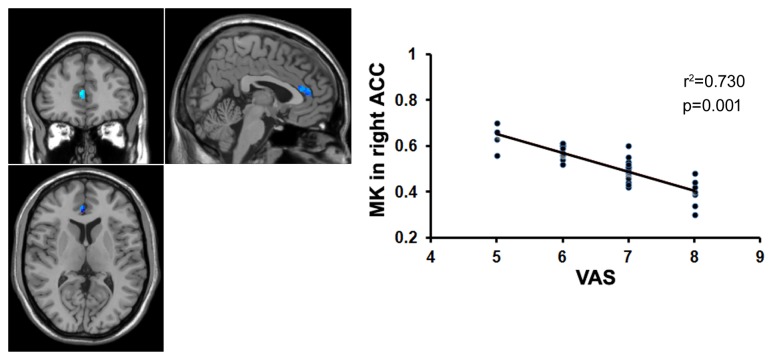
**Correlation analysis between the AK value and the VAS score.** There was significant negative correlation between the AK value in the right mPFC and the VAS score in patients with chronic myofascial pain.

To examine whether the microstructural changes in brain gray matter were associated with pain duration, VAS scores and age in the MTrPs subjects, those brain areas displaying lower AK, MK, and RK values were extracted as the ROIs to facilitate the correlation analysis between pain durations, VAS scores and age. The results revealed that in the MTrPs subjects, the MK values in the right ACC displayed significant negative correlations with the pain durations (**Figure 4**), whereas the AK values in the right mPFC and MK values in the right ACC exhibited significant negative correlations with the VAS scores (**Figures 5** and **6**). However, the MK, AK and RK values displayed no significant correlations with the age. In addition, the VAS scores exhibited no significant correlations with the pain durations, and the age also was not significantly correlated with VAS scores.

## Discussion

Although the underlying mechanisms of microstructural changes in the brain gray matter of patients with chronic pain remain unclear, neuroimaging studies have demonstrated an apparent reduction in the density or volume of these patients’ gray matter (Obermann et al., 2013; Wilcox et al., 2015; Fritz et al., 2016; Krause et al., 2016). One report even showed that the degree of reduction is compatible with a natural decline occurring over 10–20 years under normal conditions (Apkarian et al., 2004). MTrPs-related pain is a common illness resulting in chronic skeletal muscle pain (Skootsky et al., 1989; Wheeler, 2004). Though controversial, a common theory argues that sustained muscle contraction leads to local hypoxia and energy depletion as well as peripheral and central sensitization, thereby causing MTrPs-associated pain (Mense, 2004; Bron and Dommertholt, 2012). In addition, persistent MTrPs may cause central sensitization as well (Mense, 1994; Kuan et al., 2007; Xu et al., 2010).

### Microstructural Abnormalities of Gray Matter

In this study, the DKI approach was employed to elucidate the gray matter alterations in MTrPs-related pain patients. This method comprises three basic parameters: MK, RK, and AK (Tabesh et al., 2011). MK represents the average diffusional kurtosis in all diffusion directions and reflects the degree of diffusion restriction of water molecules. AK refers to the kurtosis in the direction of the diffusion eigenvector with the largest diffusion eigenvalue and mainly reflects the diffusion information along axons. RK refers to the average of kurtosis indirections perpendicular to the eigenvector with the largest eigenvalues and mainly reflects the diffusion information in the direction perpendicular to axons. The advantage of MK is that it does not depend on the spatial locations of tissues; thus, this parameter can better depict the microstructural changes of gray matter. Specifically, an increase in the value of MK reflects an increase of basal dendrites, cell density, synaptic refinement, and reactive astrogliosis (Hui et al., 2008; Cheung et al., 2009; Zhuo et al., 2012), whereas a reduced value represents microstructural injury, including neuronal edema, membrane structural damage, and myelin injury (Gong et al., 2013; Zhang et al., 2013, 2016). In addition, decreased RK and AK values reveal the decreased compactness of myelination and fiber as well as diminished myelin integrity (Wu and Cheung, 2010). Using this method, a recent study revealed that patients with chronic postherpetic neuralgia exhibited microstructural abnormalities in the gray matter, and these lesions were distributed in the right thalamus, parahippocampal gyrus, caudate and cerebellum anterior lobe, left occipital lobe and middle frontal gyrus, bilateral superior temporal gyrus and insula (Zhang et al., 2016). We observed that in comparison with the healthy controls, the MTrPs subjects displayed significantly lower MK values in the right thalamus, ACC, PCC, superior frontal gyrus, middle temporal gyrus, and left parahippocampal gyrus; significantly lower AK values in the right inferior frontal gyrus, medial prefrontal cortex, parahippocampal gyrus, caudate nucleus, and bilateral insula; and significantly lower RK values in the left lingual gyrus, temporal gyrus, right middle frontal gyrus, and bilateral ACC. The results of this study indicated that in subjects with MTrPs-related pain, these brain areas may have damaged gray matter microstructures.

Researchers have used the theory of neuronal plasticity to explain brain structural abnormalities in patients with chronic pain diseases (Woolf and Salter, 2000). According to this theory, peripheral pain stimuli are continuously delivered to the central nervous system, augmenting cortical excitability and causing the neurons in the corresponding brain areas to undergo reconstruction in response to persistent, noxious stimuli. These events may, in turn, lead to microstructural abnormalities in patients’ gray matter. Hence, we speculated that the protracted MTrPs-related pain stimuli, which induced changes in central plasticity and generated damage, was the potential cause of microstructural alterations of the gray matter in MTrPs patients.

This study revealed that the brain areas exhibiting decreased DKI parameters in MTrPs subjects were mainly concentrated in the limbic system (i.e., cingulate gyrus, parahippocampal gyrus, insula, and thalamus). The limbic system is closely related to emotional reactions to pain (Koga et al., 2015). It has been speculated that myofascial pain might be related to stressful responses of the body (Gameiro et al., 2006), which facilitates the generation of local MTrPs and pain by skeletal muscle; in turn, pain augments the stressful response. Emotional stress such as anxiety is common in patients with chronic myofascial pain (Healy et al., 2015). It is currently accepted that functional disorders in the limbic system may play an important role in the occurrence and progression of MTrPs and local pain (Srbely et al., 2010; Shah et al., 2015). The possible underlying mechanism could involve both the activation of the thalamus and the preferential activation of the limbic system by pain signals, which would thereby regulate skeletal muscle pain and the resulting emotion or mood (Svensson et al., 1997). Our results, along with those of previous studies and hypotheses, indicated that the gray matter of the limbic system is prone to damage in patients with chronic MTrPs-related pain, and that these abnormalities may be pertinent to patients’ pain perception and emotional changes. Moreover, Recent investigations have indicated that the ACC may be involved in the processes of pain and emotion (Vogt, 2005) and that other cerebral areas, such as the hypothalamus and the amygdaloid nucleus, may play an important role in regulating emotion (Blouin et al., 2013; Long et al., 2016). However, decreased DKI parameters in the hypothalamus and the amygdaloid nucleus were not observed in our experiments. This result is not understood clearly and remains open to further investigation for better understanding.

Furthermore, event-related functional MR imaging studies have revealed that pain stimuli can specifically excite some areas within the cerebrum, including the insula, primary and secondary sensory cortexes (SI, SII), ACC, frontal cortex, and thalamus (Mouraux et al., 2011; Garcia-Larrea and Peyron, 2013). These areas are referred to as the pain matrix. Niddam et al. (2008) also reported that patients with MTrPs-related pain exhibit elevated activity in SI, SII, and some forebrain islands. In our study, the main brain areas that exhibited gray matter abnormalities in the myofascial pain patients were the ACC, mPFC, insula, and thalamus. These areas are all situated within the pain matrix, which is closely associated with pain perception. Our results revealed that the microstructural alterations in the brain resulting from chronic myofascial pain are not limited to a single pain center; instead, these alterations are widely disseminated in the cerebral cortex.

### Correlation Analysis

Protracted pain can cause changes in the brain structure (Kuchinad et al., 2007; Li et al., 2016), and the underlying mechanism may be explained by neuronal plasticity (Woolf and Salter, 2000). Our data revealed that the MK values in the right ACC were significantly negatively correlated with pain duration and VAS scores, whereas the AK values in the right mPFC were significantly negatively correlated with the VAS scores. The ACC and mPFC are mainly involved in the multifunctional integration associated with pain, including pain perception and attention, emotional responses to pain, pain intensity, and anticipation of pain (Alkire et al., 2004; Ji et al., 2010). It has been reported that in patients with trigeminal nerve pain, the volume of the gray matter in the ACC is negatively correlated with the course of the disease (Obermann et al., 2013); in patients with central post-stroke pain (CPSP), the volume of the gray matter in the prefrontal cortex is negatively correlated with pain intensity (Krause et al., 2016); and in patients with chronic low back pain, the volume of the gray matter in the prefrontal cortex and AAC is negatively correlated with pain intensity (Fritz et al., 2016). Here, our data indicated that the course of the disease and pain intensity play crucial roles in the microstructural alterations of gray matter in patients with MTrPs-related chronic pain, and the gray matter microstructures in the ACC and mPFC are more vulnerable to these impacts. Recently, some investigators who analyzed the post-mortem tissue from animals found that the ACC did participate in regulating pain (Okine et al., 2016). Therefore, in order to verify whether ACC and mPFC have determinant effects on the process of myofascial pain, pathologic examinations on the post-mortem tissue from patients should be conducted in future study.

### Limitations

This study had some limitations. First, we only observed the microstructural abnormalities of gray matter in patients prior to treatment, leading to uncertainty regarding how the MTrPs-related microstructures would evolve after treatment. In other words, it is unclear whether an effective treatment may reverse the microstructural alterations in the gray matter observed in pre-treatment. In addition, it remains unclear whether the microstructural abnormalities in the gray matter results from, or resulted in, MTrPs. This point must be investigated further.

## Conclusion

Our results revealed that in patients with MTrPs-related chronic pain, the gray matter displayed microstructural alterations, which were mainly distributed in the limbic system and the pain matrix-associated brain areas. In addition, the microstructural abnormalities of gray matter in the ACC and mPFC displayed significant negative correlations with the course of the disease and pain intensity of the patients. Our results indicated that prompt and effective treatments should be provided to patients with myofascial pain, thereby preventing the MTrPs-generated noxious stimuli from entering the nerve center. Thus, we should take into consideration the mechanism of peripheral and central sensitization when developing an effective therapeutic treatment. In other words, when treating patients with pain due to central sensitization, we should formulate specific treatment strategies to normalize or alleviate the central sensitization (Nijs et al., 2010), and to account for the protection of brain tissue in order to “cure” brain microstructures as much as possible.

## Author Contributions

Conceived and designed the experiments: HZ, TY, and PX. Performed the experiments: PX, BQ, GS, and YZ. Analyzed the data: PX, BQ, GS, YZ, SC, TZ, JY, JjW, and JW. Wrote the paper: PX, HZ, and XZ.

## Conflict of Interest Statement

The authors declare that the research was conducted in the absence of any commercial or financial relationships that could be construed as a potential conflict of interest.

## Footnotes

1 http://www.mathworks.com
