# Corrigendum: Microstructural Abnormalities Were Found in Brain Gray Matter from Patients with Chronic Myofascial Pain

**DOI:** 10.3389/fnana.2017.00062

**Published:** 2017-07-21

**Authors:** Peng Xie, Bangyong Qin, Ganjun Song, Yi Zhang, Song Cao, Jin Yu, Jianjiang Wu, Jiang Wang, Tijiang Zhang, Xiaoming Zhang, Tian Yu, Hong Zheng

**Affiliations:** ^1^Department of Anesthesiology, The First Affiliated Hospital of Xinjiang Medical University Urumqi, China; ^2^Department of Anesthesiology, Zunyi Medical University Zunyi, China; ^3^Department of Radiology, Zunyi Medical University Zunyi, China; ^4^Guizhou Key Laboratory of Anesthesia and Organ Protection, Zunyi Medical University Zunyi, China; ^5^Department of Anatomy and Cell Biology, University of Kansas Medical Center Kansas City, KS, United States

**Keywords:** myofascial trigger points, chronic pain, gray matter, microstructural abnormalities, diffusion kurtosis imaging

In the original article, there was a mistake in the legends for Figures 5, 6 respectively as published. The order of the legends for Figures 5, 6 was reversed; that is, the legends for Figures 5, 6 should be switched. The correct legend appears below. The authors apologize for this error and state that this does not change the scientific conclusions of the article in any way.

**Figure 5:**
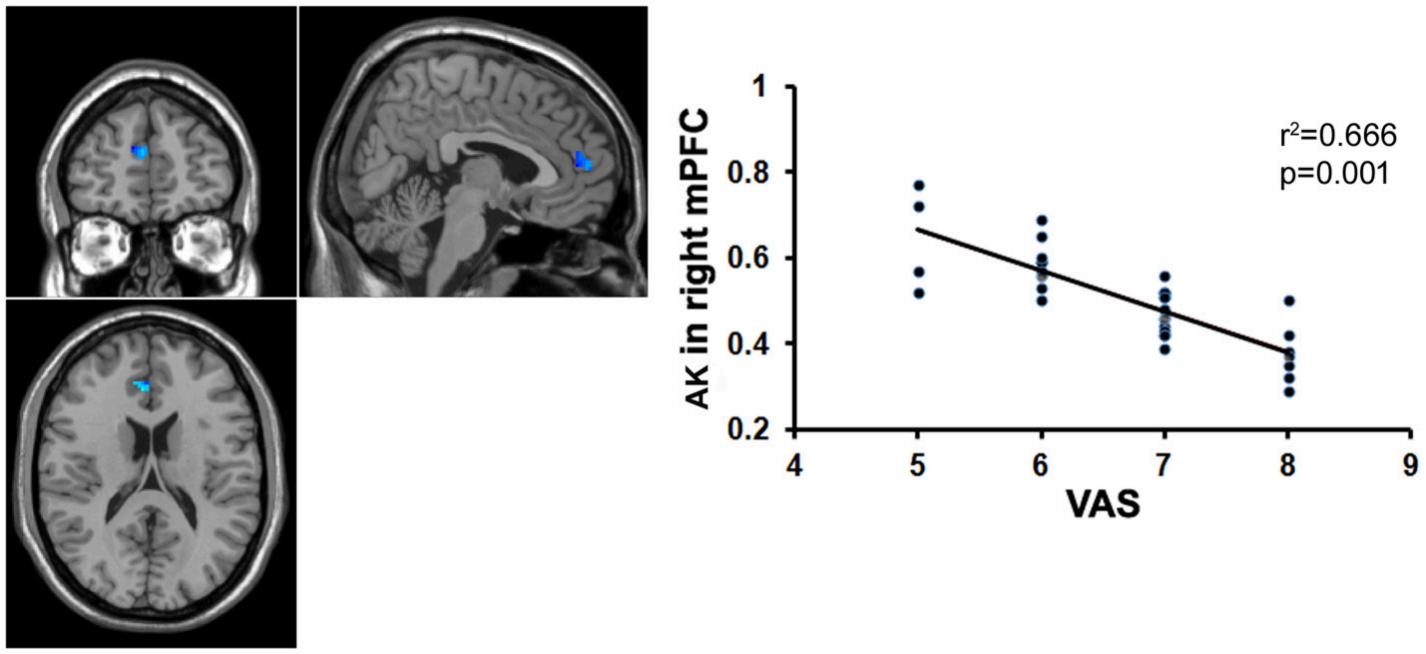
Correlation, analysis between the AK value and the VAS score. There was significant negative correlation between the AK value in the right mPFC and the VAS score in patients with chronic myofascial pain.

**Figure 6:**
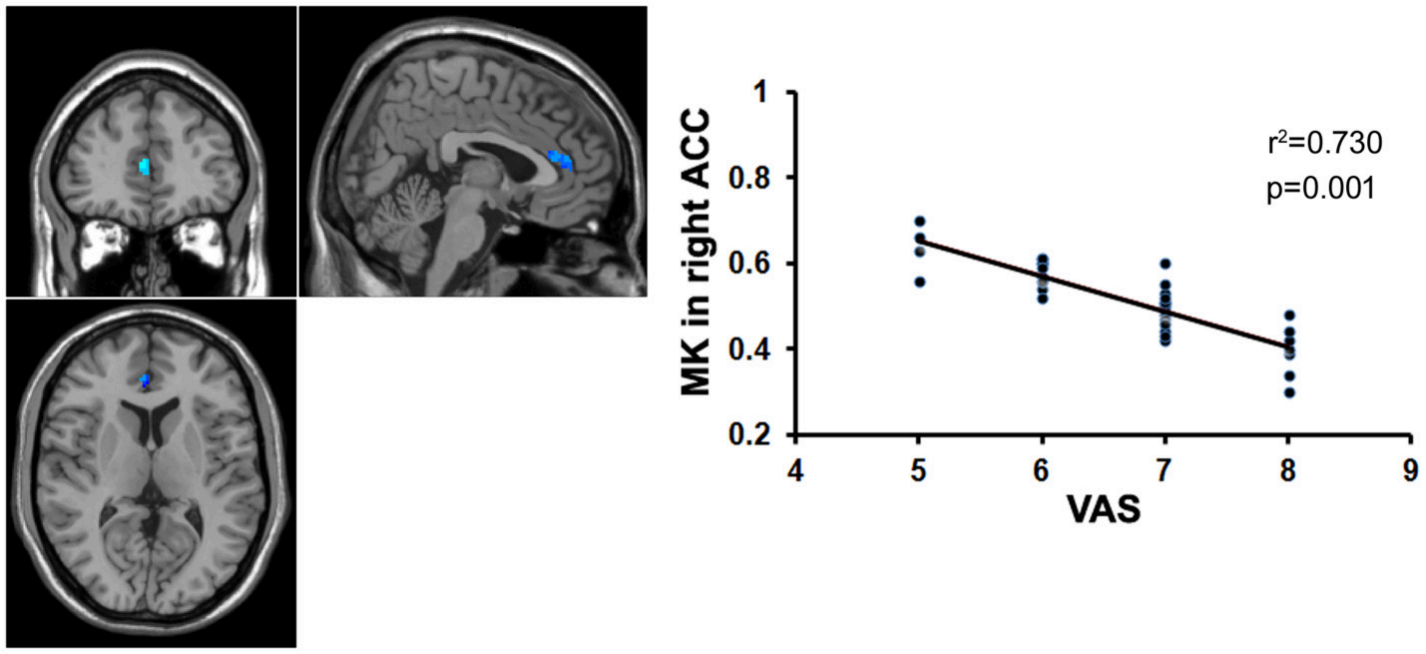
Correlation analysis between the MK value and the VAS score. There was significant negative correlation between the MK value in the right ACC and the VAS score in patients with chronic myofascial pain.

## Conflict of interest statement

The authors declare that the research was conducted in the absence of any commercial or financial relationships that could be construed as a potential conflict of interest.

